# Mechanisms of neuronal dysfunction in HIV-associated neurocognitive disorders

**DOI:** 10.1007/s00018-021-03785-y

**Published:** 2021-02-13

**Authors:** Elena Irollo, Jared Luchetta, Chunta Ho, Bradley Nash, Olimpia Meucci

**Affiliations:** 1 Department of Pharmacology and Physiology, Drexel University College of Medicine, 245 N. 15th Street, Philadelphia, PA 19102 USA; 2 Department of Microbiology and Immunology, Drexel University College of Medicine, 245 N. 15th Street, Philadelphia, PA 19102 USA; 3 Center for Neuroimmunology and CNS Therapeutics, Institute for Molecular Medicine and Infectious Disease, Drexel University College of Medicine, 245 N. 15th Street, Philadelphia, PA 19102 USA

**Keywords:** HAND, Dendritic spines, Neuronal connectivity, Neuroinflammation, Drug abuse, Cognitive impairment

## Abstract

HIV-associated neurocognitive disorder (HAND) is characterized by cognitive and behavioral deficits in people living with HIV. HAND is still common in patients that take antiretroviral therapies, although they tend to present with less severe symptoms. The continued prevalence of HAND in treated patients is a major therapeutic challenge, as even minor cognitive impairment decreases patient’s quality of life. Therefore, modern HAND research aims to broaden our understanding of the mechanisms that drive cognitive impairment in people with HIV and identify promising molecular pathways and targets that could be exploited therapeutically. Recent studies suggest that HAND in treated patients is at least partially induced by subtle synaptodendritic damage and disruption of neuronal networks in brain areas that mediate learning, memory, and executive functions. Although the causes of subtle neuronal dysfunction are varied, reversing synaptodendritic damage in animal models restores cognitive function and thus highlights a promising therapeutic approach. In this review, we examine evidence of synaptodendritic damage and disrupted neuronal connectivity in HAND from clinical neuroimaging and neuropathology studies and discuss studies in HAND models that define structural and functional impairment of neurotransmission. Then, we report molecular pathways, mechanisms, and comorbidities involved in this neuronal dysfunction, discuss new approaches to reverse neuronal damage, and highlight current gaps in knowledge. Continued research on the manifestation and mechanisms of synaptic injury and network dysfunction in HAND patients and experimental models will be critical if we are to develop safe and effective therapies that reverse subtle neuropathology and cognitive impairment.

## Introduction

Human immunodeficiency virus (HIV) infection can cause cognitive, motor, and sensory deficits that are collectively termed HIV-associated neurocognitive disorders (HAND) [1]. Typical symptoms of HAND include loss of attention, concentration, and memory, reduced motivation, irritability, depression, and slowed movements [1]. Symptom severity can range from asymptomatic neurocognitive impairment to HIV-associated dementia in the most severe cases [2]. HIV infection has been transformed into a chronically manageable disease by combined antiretroviral therapy (ART) [1, 3]. ART effectively controls HIV replication and sharply reduced the incidence of HIV-associated dementia, but it does not entirely prevent the development of HAND [4, 5]. Mild neurocognitive symptoms persist in roughly 50% of patients on ART [6] and only a small fraction of this group develops severe forms of HAND [1]. In addition, the onset or progression of HAND may be worsened in aging patients [7] and is exacerbated by common comorbidities like drug abuse [8]. There are no effective treatments for HAND despite extensive studies, suggesting that there is still much to learn about the pathogenic mechanisms underlying the disorder.

The neuropathology of HAND has also been drastically changed by ART. Early in the HIV epidemic, many patients presented with encephalitis and neuronal loss, which were associated with severe forms of dementia [3, 9]. Today, ART-treated patients with HAND do not present with neuronal loss or overt neuropathology, suggesting that more subtle neuronal changes drive the pathology [10]. This likely includes simplification of neuronal networks and loss of dendritic spines in brain regions that are necessary for learning and memory processes [3]. Synaptodendritic damage can directly relate to aberrant neuronal circuitry and impaired cognitive functions, and it is associated with disease progression [11].

Clinical studies of HAND patients are important for setting research directions, but the number of clinical samples available for analysis has declined since patients on ART now live close to a normal lifespan. However, new non-invasive neuroimaging techniques have become a useful alternative approach. Despite their focus on functional alterations, neuroimaging techniques can provide some information about structural changes and, therefore, aid investigations on the brain in virally suppressed subjects. Neuroimaging can also reveal insights about other factors relevant to the pathology, such as inflammatory markers and select pathogenic proteins [12, 13]. These studies, in combination with insights gained from pre-clinical model systems, suggest that HAND is brought on or worsened by subtle synaptodendritic damage in select brain regions, and by extension, disruption of neuronal networks that are involved in learning and memory processes. This review highlights our current knowledge of synaptodendritic damage and neuronal dysfunction in HAND from clinical and pre-clinical studies, and suggests new insights that could lead to effective HAND therapies. We begin by discussing neuroimaging and neuropathology studies of human HAND patients that show the structural, metabolic, and functional alterations in the HIV-infected brain. Next, we cover studies from animal and in vitro models that provide more precise and specific insights on region-specific synaptodendritic damage that are not available from human neuroimaging studies. Finally, we review the molecular mechanisms that are currently thought to promote synaptodendritic damage and neuronal dysfunction, and highlight some unanswered questions and future directions of HAND research that may lead to new therapeutic approaches.

## Neuroimaging and neuropathological studies in HAND patients

Neuroimaging methods have allowed us to observe important aspects of HAND pathology in today’s patients, including brain volume, neuroinflammation, and functional connectivity [14]. However, neuroimaging approaches generally lack the resolution to detect changes in small structural components of neurons like synapses and dendritic spines, and so far, they detect few surrogate measures of inflammation. Despite these limitations, neuroimaging studies provide much needed context on modern HAND in the absence of neuropathology studies of treated patients with well-controlled infection. By considering the findings and limitations from both neuroimaging and neuropathology studies, we can better understand how HAND pathology has changed in treated patients as well as guide future studies that seek to understand the pathological drivers of HAND in the ART era.

Magnetic resonance imaging (MRI) is often the initial step in the diagnostic approach for HAND, although to date, there is no unique neuroimaging technique capable of confirming a HAND diagnosis [15]. Published MRI studies of HAND patients show various alterations in brain volume and suggest that these changes may contribute to cognitive impairment [16, 17]. A recent study used functional MRI to measure brain atrophy in aviremic ART-treated people with HIV compared to uninfected controls over a period of 2 years [18]. The HIV group showed reduced cortical thickness and subcortical brain volumes and poorer cognitive performance compared with controls, but the changes in cognitive performance and brain volumes during the 2-year period were similar between the groups. Another clinical study reported that thinning of cortical areas including the frontal and temporal cortices was associated with nadir CD4 count in patients with controlled infection [19]. Together, these studies suggest that maintaining full viral suppression can minimize brain injury in people with HIV, but subtle, region-specific brain damage may persist from before initiation of ART. Similar neuroimaging studies have examined changes in gray and white matter of patients on ART. For example, one group reported widespread changes in gray matter volume in patients with controlled infection, and the volume of specific brain regions correlated with impairment in specific cognitive tasks [20]. Another recent study of virally suppressed patients reported that white matter alterations were present in patients with HAND, but HIV patients without cognitive impairment had similar white matter measures compared to uninfected controls [21]. Furthermore, more pronounced white matter alterations were observed in older patients with HIV [22], which may reflect accelerated brain aging. White matter alterations are also associated with impairment of several cognitive domains in patients with controlled infection and no other medical comorbidities [23]. The changes in brain volume described here may also reflect synaptodendritic injury, but this must be confirmed by neuropathology studies of patients with controlled infection [24].

Resting-state functional MRI can detect changes in brain connectivity in people with HIV and can help to determine how altered neuronal network activity contributes to cognitive impairment. One recent study found abnormal connectivity patterns at a global as well as a regional scale in subjects with HAND compared to age-matched controls, primarily in portions of the lateral occipital cortex and the cingulate cortex [25]. Their results showed that a combination of network analysis and graph theoretic methods can help to better understand the mechanisms of neurologic diseases and identify diagnostic biomarkers for HIV-related neurological damage. A smaller clinical study of patients with controlled infection reported changes in salience and executive networks that correlated with neuropsychological test scores [26]. Other evidence suggests that during acute HIV infection, changes in resting-state functional connectivity in several brain regions correlate with depressive symptoms [27], and switching ART regimens may affect functional connectivity in asymptomatic patients [28]. Thus, functional connectivity appears to be altered in HAND patients with controlled infection and may correlate with cognitive impairment as well as other auxiliary symptoms of HAND.

Sustained subtle neuroinflammation is also thought to contribute to synaptodendritic damage and neuronal dysfunction in the ART era. Several groups have studied correlates of neuroinflammation in people with HIV, most notably using positron emission tomography (PET) imaging of translocator protein (TSPO) [29–31]. TSPO is a mitochondrial membrane protein that is strongly expressed in glial cells and is upregulated during periods of neuronal injury or neuroinflammation [32]. Although measuring TSPO with PET ligands is a common approach in studies on neuroinflammation, recent work suggests that the physiological functions of TSPO are not completely understood and TSPO ligand binding may not directly translate across species [33]. Therefore, although TSPO imaging is widely used, results from these studies should be considered with caution and in consideration with other approaches when possible. Overall, TSPO studies of HAND patients found correlations between certain cognitive domains and tracer uptake in various brain regions, but their results vary as they used different patient populations and different criteria for defining the degree of cognitive impairment [34]. Another approach to measure correlates of neuroinflammation in patients involves diffusion basis spectrum imaging (DBSI), which can separate measures of inflammation-induced cellularity and white matter integrity [35]. For example, one DBSI study of a cohort of aviremic people with HIV showed that they had elevated brain cellularity compared to HIV negative controls, and younger people with HIV showed the greatest cellularity changes [36]. However, when they adjusted for duration of ART, the association between age and cellularity in HIV + individuals was no longer significant, suggesting that prolonged treatment reduces brain inflammation.

As non-invasive imaging approaches are increasingly necessary to understand HAND in the ART era, future neuroimaging studies are positioned to play important roles in uncovering brain region and network-level alterations in HAND patients with controlled infections.

Neuropathology studies are currently necessary to confirm synaptodendritic damage and neuronal alterations in relevant brain regions. Unfortunately, most published neuropathology studies on HAND are from the pre-ART era or use clinical samples from patients with acquired immunodeficiency syndrome (AIDS), suggesting that they are not completely reflective of the current pathology [37]. Therefore, new studies using tissues from ART-treated patients with controlled infection are urgently needed. However, published neuropathology studies do provide insight on features of HAND and evidence that synaptodendritic damage and altered neurotransmission play important roles in HAND.

Gene expression studies of human brain tissue offer an overhead view of many interconnected systems and allow measurement of synaptic and neuronal genes alongside of other genes that are thought to contribute to synaptic injury, including those related to inflammation and bioenergetics. Older studies from patients with moderate-to-severe HAND generally report an upregulated brain immune response and a downregulation of synaptic transmission [38]. These findings appear less severe in milder forms of HAND as patients without HIV encephalitis but with sustained neurocognitive impairment show lower brain HIV levels, weaker immune responses, and fewer neuronal pathway changes in the neocortex [39]. Though there are still some contradicting data, alteration of inflammatory genes is a common feature of HAND, ranging from widespread, uncontrolled inflammation and tissue damage in HIV-encephalitis tissue, to induction of interferons, cytokines, and tissue injury in neurocognitively normal HIV patients [40]. Furthermore, white matter alterations in the expression of chemokines, cytokines, and β-defensins correlate with cognitive impairment in patients without HIV encephalitis [40]. A recent gene expression study also supports inflammatory dysregulation in older HAND patients on ART, as brain tissue from these patients showed upregulation of genes related to interferon signaling, stress, and immune responses, as well as decreased expression of genes involved in cellular metabolism and myelin production [41]. Overall, these studies shed light on some of the underlying changes contributing to HAND in patients with more controlled infection and further support the hypothesis that subtle neuronal disruption is an important component of modern HAND.

Several early neuropathology studies identified synaptodendritic damage in specific brain regions as a correlate of cognitive impairment in patients with AIDS. One of the early reports showed that neocortical dendritic and presynaptic damage was a strong correlate of neurocognitive impairment [42]. Another study reported reduced branching density, dendritic spines, and other types of dendritic simplification throughout the hippocampus [43]. Presynaptic damage was also found in the hippocampus and putamen of AIDS patients [44], and this study showed that a combined index of both synaptic and dendritic injury markers was more strongly associated with global neuropsychological impairment ratings than either marker alone. Notably, dendritic injury was brain region specific [40]. Additional studies have shown that synaptodendritic degenerative changes in HIV correlate with the presence and severity of cognitive impairment [3, 45]. As these studies all found an association between synaptodendritic injury and cognitive impairment in tissue from HAND patients, it remains possible that cognitive impairment in treated patients with controlled infection may be the result of more subtle forms of synaptic injury or functional alterations in neurons. Indeed, a small gene expression study of brain tissue from treated people with HIV revealed no changes in synaptic markers in neurocognitively normal patients, but increased expression of immune response genes and the anti-inflammatory gene heme oxygenase 1 [24]. This study provides some evidence that preserving synapses could be an approach to treat HAND, even in an environment of subtle, chronic brain inflammation. However, this claim will require additional validation in future studies.

One mechanism that may lead to synaptodendritic damage in HAND patients is the accumulation of neurotoxic amyloid-β, especially as patients on ART become older. Studies of brain samples from HIV patients have shown altered amyloid precursor protein processing and the presence of amyloid-β plaques and oligomers [46–49]. One study showed that patients with HIV encephalitis had more intraneuronal amyloid-β accumulation compared to HIV cases with no significant pathology [48], and another study supported intracellular amyloid-β deposition in brain samples from HAND patients and showed parenchymal amyloid-β deposition in samples from older patients exposed to ART [46]. The latter authors suggested that local inflammatory responses to HIV in the brain could increase amyloid precursor protein production and susceptibility to amyloid deposition, and ART itself may contribute to an overall increase in amyloid deposition [46]. Interestingly, these studies suggest a distinct type of amyloid-β pathology in some patients with HAND that differs from Alzheimer’s disease. While Alzheimer’s disease is characterized by neuritic, extracellular amyloid plaques, studies of HAND tissue show diffuse and intracellular expression of amyloid-β that may be a precursor to neuritic plaques [46, 50, 51]. However, more recent PET imaging studies measured amyloid deposition in the brains of living HAND patients and did not show amyloid-β accumulation [52–55], and a recent neuroimaging study reported no change in amyloid levels in virally suppressed patients [56]. The conflicting results from these studies may be due to different factors. For example, amyloid dysregulation may be absent or mild in treated patients with less severe neuroinflammation, or amyloid pathology may only be relevant in patients with specific genotypes [57]. However, amyloid pathology may become more relevant as the HAND patient population on ART ages. Therefore, additional studies are required to determine if amyloid pathology or toxic amyloid oligomers contribute to HAND in treated patients.

Several studies suggest that neurotransmitter systems are disturbed in HAND patient samples. GABAergic markers in brain samples from HIV-infected patients with and without HAND appear to be significantly decreased in most areas of cerebral neocortex, the neostriatum, and the cerebellum [58]. These findings were correlated with neuroimmune markers in the brain and mRNA expression of neuromodulators including dopamine receptor type 2 and the opioid neurotransmitter preproenkephalin, which were also reported to be dysregulated in a previous study of the same patient cohort [59]. Thus, dysfunction of inhibitory neurotransmission may contribute to HAND, and this could possibly result from subtle, ongoing brain inflammation and/or changes in other neuromodulators. Altered inhibitory neurotransmission in HAND patients may also be due to the breakdown of perineuronal nets, a type of extracellular matrix that stabilizes inhibitory synapses from parvalbumin interneurons [60]. Of note, increased expression of matrix metalloproteinases that degrade perineuronal net components has been reported in brain tissue from people with HIV on ART [61]. Older studies have also reported HIV-induced upregulation of matrix metalloproteases in the CNS, in particular MMP-2 [62], which was linked with HIV-associated dementia [63].

Dysregulated inhibitory circuits may also lead to abnormal excitatory neurotransmission via the glutamate system, which has long been reported in HAND patients and models [64]. However, clinical studies of brain glutamate levels in HAND have reported conflicting results, which likely reflects the underlying complexity of HAND in different patients [64]. While there is evidence that ART can decrease extracellular glutamate levels in the brain [65], many studies in model systems have demonstrated that HIV proteins may directly and indirectly enhance glutamatergic signaling [66, 67]. Excitatory and inhibitory neurotransmission are likely dysregulated via multiple pathways in HAND, and this may also include dysregulation of other neurotransmitter systems. For instance, dysfunction of dopamine signaling in HAND is an ongoing area of research. Although some evidence suggests that HIV neurotoxins may directly modulate the dopamine transporter and damage brain areas with high dopamine expression [68], drug abuse is another common comorbidity that can transiently elevate dopamine levels in HAND patients [69]. This can lead to several outcomes, including circuit adaptations and neuroinflammation in dopamine-rich regions [70], which may further contribute to synaptodendritic damage. Future studies that explore both excitatory and inhibitory systems in parallel as well as the status of other neuromodulators may help to provide a more integrated picture of neurotransmitter systems in the presence of HIV or other related factors.

Clinical studies of HAND provide evidence that well-controlled HIV infection can still dysregulate neuronal functions in specific brain regions, suggesting a potential underlying cause of cognitive impairment in treated patients. However, it is still not entirely clear how controlled HIV infection contributes to this more subtle and region-specific neuronal dysfunction. Though some clinical studies of HAND would surely benefit from updates with treated patients, the field has expanded on insights gained from current clinical research using a variety of pre-clinical models. As discussed in the next section, these models support synaptodendritic injury as a substrate of neurocognitive impairment in HAND.

## Synapodendritic damage and neuronal activity changes in HAND experimental models

In vitro and in vivo models of HAND have provided important insights into the neuropathological features of HAND that may exist in treated patients. Although these models have their own set of limitations, they allow for much more precise examination of neuronal structure and function and how specific HIV proteins and related stimuli contribute to synaptodendritic damage and alter the activity of neuronal networks. This section reviews recent findings from model systems regarding changes in synapses, dendritic spines, and electrophysiology.

Excitatory synapses are commonly studied through quantification and morphometric analyses of dendritic spines, which are small protrusions along the dendrite that form the post-synaptic component of most excitatory synapses [71, 72]. Dendritic spines exist along a continuum of morphologies that is often classified into groups, including the more mature mushroom and thin spines, and the immature stubby spines and filopodia [73]. Several studies over the past 2 decades have shown that spine morphology is correlated with function, as these structural changes allow for variable abundance of glutamate receptors as well as key components of the post-synaptic density, thus dictating synaptic strength (many excellent reviews have been published on this important topic; the readers are referred to some of the most recent papers, e.g., [73–75]). Thus, the shape of a spine also reflects its stability or maturation as a synaptic site. For instance, mushroom spines, which have a large bulbous head and a narrow neck, form the most stable synapses [76]. Their large head contains multiple AMPA and NMDA receptors, and their narrow neck provides a well-controlled biochemical microenvironment [77]. Thin spines, which have a smaller head attached to a narrow neck, often form more transient synapses [78]. As expected, these spines also have lower expression of glutamate receptors and other components of the post-synaptic density [79]. In the adult cortex and hippocampus, more than 65% of dendritic spines are thin spines, while just 25% are mushroom spines [80], highlighting the vast neuroplasticity in these regions. Stubby spines lack a neck region and presumably the ability to buffer synaptic ion flux, and are prevalent during early postnatal development [80]. Filopodia are hair-like structures that may act as precursors to thin dendritic spines. Filopodia and stubby spines account for approximately 10% of spines in adults [80]. Dendritic spines are highly enriched in filamentous actin and are highly motile in response to synaptic activity [81]. Therefore, spines are dynamic structures that can change their shape, even throughout adulthood [82]. However, the plasticity of dendritic spines also makes them vulnerable to neurotoxins and inflammatory mediators that impair neuronal functions.

Different groups, including ours, have reported how dendritic spines may be altered in HAND by studying HIV-transgenic rats. These rats provide a similar environment to HIV-infected patients on ART, as they express viral proteins but do not produce infectious virus [83]. The HIV-transgenic rat also presents with cognitive impairment, including deficits in executive function [84, 85], temporal processing and long-term episodic memory [86], and spatial learning [87, 88]. Notably, these rats have impaired cognitive flexibility, or the ability to shift strategies in response to different demands. This requires neuronal activity in the medial prefrontal cortex [89] and male HIV-transgenic rats present dendritic spine deficits on layer II/III pyramidal neurons in this region [90], including a reduced density of thin and mushroom spines and increased density of stubby spines [84]. As predicted for highly plastic spines, the density of mature thin spines is more strongly associated with cognitive flexibility than any other spine type [84]. Furthermore, HIV-transgenic rats show dendritic spine and branching deficits in layer II/III neurons of the motor cortex, including an increased percentage of filopodia [37], suggesting that this could correlate with motor dysfunction. Another study examined synaptodendritic injury in older female and male HIV-transgenic rats and reported thin spine deficits and increased stubby spine density on similar regions of proximal dendrites, although neurocognitive impairment later in disease progression predominantly affected male rats [91]. The same group reported that in animals of a similar age, methylphenidate caused a significant shift toward increased frequency of thin spines on lower order dendrite branches [92]. Dendritic spine deficits in the HIV-transgenic rat likely involve multiple factors in addition to expression of HIV proteins, as chronic immune activation is typically observed in these animals [93–95], which could drive synaptodendritic damage.

Synaptic injury can also be caused by individual HIV proteins, including HIV tat and gp120. Tat is present in the cerebrospinal fluid of virally suppressed patients [96] and has multiple direct and neuroinflammatory effects that are considered key contributors to HAND neuropathology [66]. There are multiple splice variants of tat, and the most studied version is a truncated tat 1–86. A recent meta-analysis uncovered that 40% of the 973 studies examining Tat used the 1–86 variant, while only 15.5% used Tat 1–101, which is the most common splice variant observed in people infected with HIV-1 subtype B [97]. This suggests that one should exercise some caution when considering studies using tat 1–86 or other truncated forms of tat. Indeed, there are functional differences reported between tat splice variants that may contribute to synaptodendritic injury in HAND, and common tat-transgenic animal models typically do not express the full-length tat 1–101 [97]. That said, tat 1–86 exposure in animal models does affect dendritic spines and neuronal function in brain regions including the hippocampus, cortex, and striatum. One study reported that tat 1–86 injection in the lateral ventricle of male mice produced spine deficits in the retrosplenial cortex, which predicted cognitive deficits in a trace fear conditioning model [98]. Another report shows that inducible tat-transgenic male mice have reduced dendritic spine density in apical dendrites of the hippocampus, which was associated with reduced LTP in the region [99]. Tat-transgenic male mice also present with damage to neurons expressing D2 dopamine receptors in the striatum, characterized by reduced dendritic spine density in the second- and third-order dendrite branches [100]. Although inducible tat-transgenic female and male mice both have dendritic spine deficits in striatal medium spiny neurons, male mice had slightly more pronounced cellular deficits as well as a motor learning impairment [101].

The HIV envelope glycoprotein gp120 can also produce dendritic spine deficits, although recently only a few studies have examined gp120 animal models—since tat is thought to have a prominent role in virally suppressed patients. However, considering that transient expression of gp120 in the first days of infection can trigger long-term events and/or neuroinflammation [102], the contribution of this viral protein should not be ignored. Indeed, past studies showed that a single injection of gp120 elicited long-term damage. Specifically, intracerebroventricular injection of gp120_IIIB_ in adult male rats decreases dendritic spine density in several cortical regions, including the medial prefrontal cortex [90] and the motor cortex [37]. As spine loss in the prefrontal cortex correlated with decreased cognitive flexibility in the same rats, it is possible that gp120-induced spine loss in the motor cortex could contribute to motor deficits. Other studies that examined female and male gp120-transgenic mice reported dendritic spine deficits throughout the hippocampus [103] and the dorsal striatum [104].

Alterations of dendritic spines and synapses are expected to be accompanied by changes in neuronal activity and connectivity. Indeed, several groups have demonstrated that the animal models discussed above display changes of neuronal activity in the same brain regions where spine deficits occur, including the prefrontal cortex, hippocampus, and striatum (Table 1). One such study of hippocampal tissue in female HIV-transgenic rats reported that dorsal CA1 pyramidal neurons were not as excitable as neurons from control animals, which could be part of a feedback mechanism to protect hippocampal neurons from excitotoxicity induced by HIV proteins [105]. On the other hand, studies in male HIV-transgenic rats showed hyper-excitability in medial prefrontal cortex layer V and VI pyramidal neurons [106], suggesting that select brain regions may respond differently to HIV neurotoxins. Similar findings were observed in brain tissue from inducible tat-transgenic male mice, as CA1 pyramidal neuron excitability was reduced, while layer II/III neurons in the medial prefrontal cortex were hyper-excitable [107]. These phenotypes may be at least partially driven by changes in inhibitory neurotransmission, as male tat-transgenic mice show reduced exocytosis of GABA in the prefrontal cortex but not the hippocampus [108]. Evidence also suggests that specific types of neurons may be more vulnerable to HIV neurotoxins. Investigations in the dorsal striatum of male inducible tat-transgenic mice revealed increased excitability, reduced dendritic spine density, and a shift toward immature spine types in medium spiny neurons expressing the D2 dopamine receptor, whereas medium spiny neurons expressing D1 dopamine receptors did not display these impairments [100]. Additionally, altered neuronal activity in HAND models can be exacerbated by comorbid drug abuse. For example, cocaine increased the excitability of pyramidal cells in the layer V and VI prefrontal cortex of male HIV-transgenic rats [109], opioids reduced GABAergic neurotransmission in the striatum of male and female mice in the presence of tat [110, 111], and methamphetamine affects synaptic and neuroplasticity markers in male and female HIV models [112, 113]. These findings are in line with the complex and multifactorial HAND scenario observed in the clinic and highlight our incomplete knowledge of the disorder.

**Table 1:** Dendritic spine density and excitatory/inhibitory balance in select brain regions of HAND/HIV models

	Cortex/PFC	Hippocampus	Striatum
Spine density	Reduced in several models [37, 84, 90, 91, 98, 118]	Reduced in several models [99, 103]	Reduced in several models [100, 101, 104, 188]
Excitatory status	Hyper-excitable in PFC of several models [106, 107, 109, 118, 190]	Decreased excitability in CA1 [105, 107] and in vitro tat models [127]	Differential effects on D1 and D2 MSNs [100]
Inhibitory status	Reduced IPSCs/GABA efflux in tat models [108, 159]	No change in GABA efflux [108], increased inhibition in vitro tat models [114, 115, 127]	Reduced IPSCs in tat model [111]

In vitro models of exposure to HIV-1 proteins or related neurotoxins generally support the findings from animal models of HAND and recently have provided new insights on how HIV neurotoxins and comorbidities disrupt neuronal networks, and how neurons respond to these insults. For example, we have recently shown that exposure to gp120_IIIB_ leads to dendritic spine deficits in cultured cortical neurons in a process that requires secretion of interleukin-1β (IL-1β) from co-cultured glial cells, suggesting that IL-1β is an important contributor to HIV-induced spine deficits in the cortex [90]. Other studies in rat hippocampal cultures have shown that gp120_IIIB_ increased inhibitory synapses and tonic inhibition, which also required gp120-induced secretion of IL-1β from microglia in the same cultures [114, 115]. Since IL-1β indirectly activated NMDA receptors in these neurons [114], the shift toward inhibitory input may represent an adaptive mechanism that could at least partially explain why hippocampal neurons are not as hyper-excitable as other types of neurons in animal models of HAND. The number of excitatory and inhibitory synapses on neurons can be scaled up or down in response to neuronal activity in a process called synaptic scaling [116], and this process appears to be a key compensatory mechanism in HAND [117].

Several recent in vitro studies have also examined how HIV tat affects synapses and neuronal excitability either alone or with comorbid factors, and all studies in this paragraph examined the truncated tat 1–86 variant. In cultures of mouse primary neurons from the prefrontal cortex, tat treatment caused dendritic spine deficits and promoted excitotoxicity in line with studies from tat-transgenic mice, but these effects were mitigated by cannabinoid receptor 1 (CB1) agonists [118]. Thus, CB1 signaling could function as a feedback mechanism to control tat-induced excitotoxicity in prefrontal cortex neurons. The same group reported that CB1 receptors are upregulated in neurons of the prelimbic prefrontal cortex in female tat-transgenic mice [119], suggesting that this system may be sex-specific. However, a new study examining cultured rat hippocampal neurons reported that tat treatment prevents CB1-mediated presynaptic inhibition at excitatory synapses, but does not affect inhibitory synapses, suggesting that CB1-mediated neuroprotection may not extend to the hippocampus [120]. On the other hand, opioids appear to enhance HIV tat effects in cultured striatal neurons, as morphine exacerbated calcium and sodium flux in medium spiny neurons exposed to tat, which contributed to dendritic spine deficits and varicosities [121]. Opioid use has previously been shown to reduce dendritic spine density in other types of neurons, including cortical [122] and hippocampal neurons [123], suggesting that comorbid opioid use may broadly dysregulate neuronal circuits in HAND patients or animal models. Importantly, drugs of abuse may also indirectly affect neuronal structure and function by modulating other non-neuronal cells in the CNS [8].

It is also possible that ART itself contributes to synaptodendritic injury, either alone or in combination with other comorbid factors. For instance, select combinations of antiretrovirals and methamphetamine can alter expression of the presynaptic marker synaptophysin in cortical cultures, although ART compounds alone seem to minimally affect synaptophysin levels, even over a prolonged exposure [124]. Reduced levels of synaptophysin protein were also found in the hippocampus of SIV-infected macaques on an ART regimen compared to SIV-infected controls [125]. Follow-up in vitro experiments indicated that select protease inhibitor antiretroviral drugs reduced synaptophysin and MAP-2 expression by promoting oxidative damage [125] and that protease inhibitors can also affect neuronal function through effects on oligodendrocytes, such as inhibition of oligodendrocyte progenitor cells maturation [126]. Administration of these drugs to mice reduced myelin expression in the frontal cortex [126]. This suggests that some ART may affect white matter volume, in line with the recent neuroimaging studies discussed above.

Most of the previously mentioned studies examined structural and functional changes in individual neurons of in vitro and in vivo models, but it is also possible to measure neuronal network activity in vitro using multielectrode arrays. A few reports have used multielectrode arrays to better understand how HIV neurotoxins alter activity at the network level in select types of primary neurons. One group examined rat primary hippocampal neurons and found that full-length tat 1–101 strongly suppressed neuronal activity and neuronal oscillations from 0 to 5 Hz, which was associated with attenuated spike frequency and amplitude across the culture. Additional tat treatments did not further reduce activity but did promote non-synchronous random firing. Neuronal activity was partially restored by removal of tat or in cultures with astrocytes, and in line with the previous studies suggesting tat promotes GABAergic inhibitory neurotransmission in the hippocampus, GABA receptor antagonists increased neuronal activity in tat-exposed cultures [127]. These results suggest that the full-length and truncated tat affect neuronal activity similarly and provide a new and important perspective on how tat modulates neuronal network activity over time. One other study used multielectrode arrays to assess the effect of HIV nef protein on rat hippocampal neurons. Neurons exposed to nef from astrocyte-derived extracellular vesicles showed a progressive reduction of neuronal activity over time with almost no network activity after 48–96 h of exposure [128]. However, the neurons in these cultures were somewhat young at 14 days in vitro, so effects on neuronal activity may not be comparable to the previous study that examined neurons at 25 days in vitro.

**Fig. 1:**
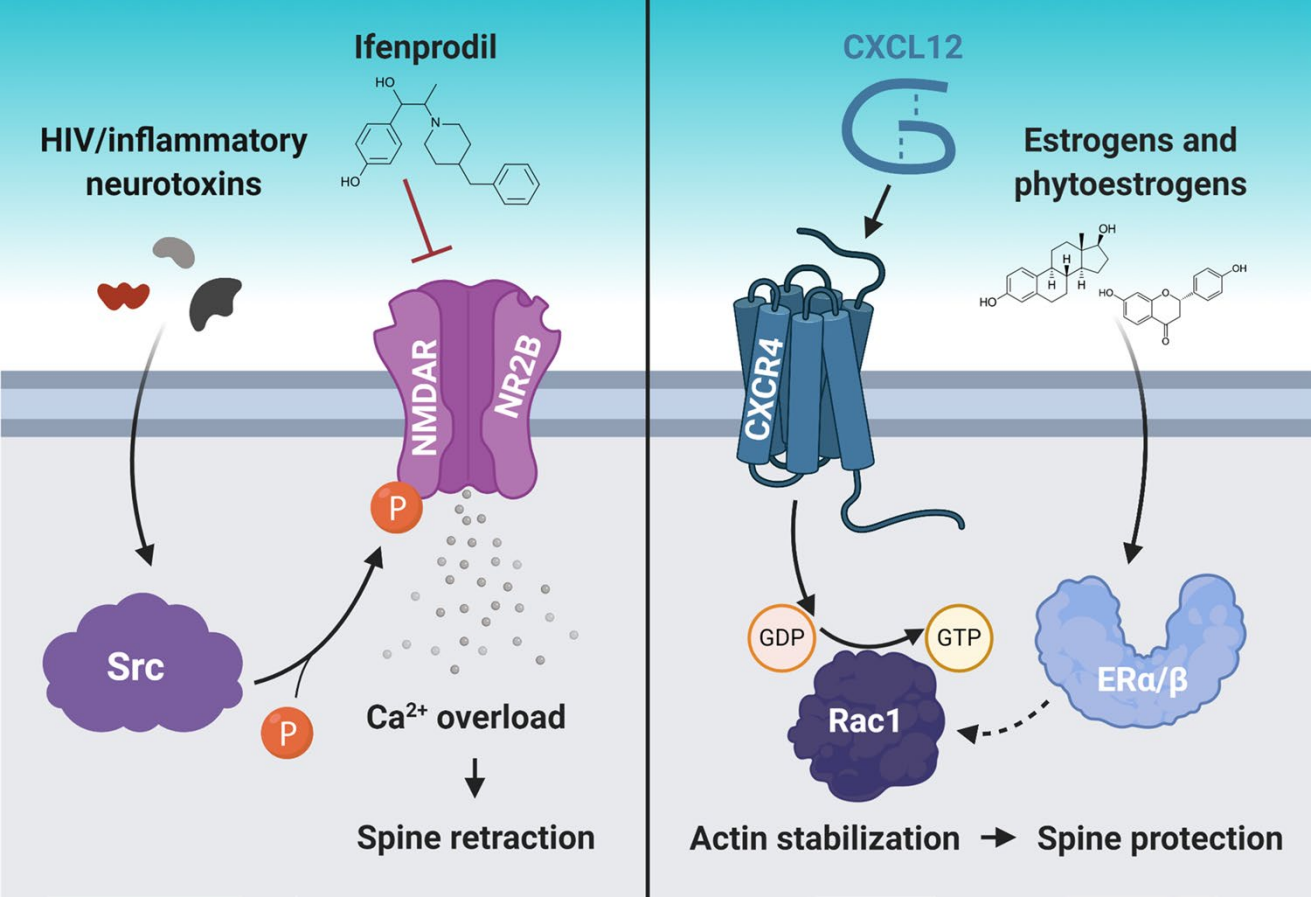
Dendritic spine loss in HAND models can be reversed by different approaches. Recent studies have reported possible approaches to reverse dendritic spine deficits in animal models of HAND, which also improved their cognitive function. These approaches involved inhibiting excess Ca^2+^ influx into neurons (left panel) and promoting neuronal actin stabilization (right panel). In the left panel, HIV neurotoxin activation of Src causes excessive Ca^2+^ influx through NR2B-containing NMDA receptors (NMDAR) in the retrosplenial cortex, but inhibiting these receptors prevents excessive Ca^2+^ influx and spine retraction in the same region. In the right panel, CXCL12 binding to CXCR4 on cortical neurons activates a Rac1-mediated pathway that stabilizes actin, a critical structural component of dendritic spines, which protects existing spines. Interestingly, CXCL12 is also able to downregulate NR2B-containing NMDARs. Moreover, various estrogen and phytoestrogen molecules can increase dendritic spine density via estrogen receptor (ERα/β) signaling, which may also involve actin stabilization via Rac1 signaling. The mechanisms presented in this figure are explained in detail in the next section

To date, most studies that examined the relationship between dendritic spines and behavior have been correlational. However, new techniques could surpass correlations and examine the downstream effects of structural and functional plasticity on the formation of new behavior. One example is activated synapse targeting photoactivatable Rac1, which allows erasure of recently activated synapses [129]. The first study using this technique suggested that learning a new motor skill relies on the formation of dense synaptic ensembles [129]. More recently, this technique helped to demonstrate that the acute anti-depressive effects of ketamine rely on neuronal activity in the prefrontal cortex. However, ketamine’s effects were only sustained upon formation of dendritic spines [130]. Several groups have found correlational relationships between dendritic spine density and neurocognitive functions in multiple animal models of HAND. These researchers attenuated dendritic spine and memory deficits by pharmacologically targeting NR2B-containing NMDA receptors [98], chemokine receptors [84], or estrogen receptors [131, 132]. While each of these studies explored different pharmacological mechanisms (Fig. 1), the downstream effects were similar—increased spine density, a shift toward more mature spine phenotype, and improved behavior. Although these experiments did not examine the network-level electrophysiology underlying recovery of spines and behavior, the structural arrangement of dendritic spines is tied to neuronal activity and, thus, altered neuronal network activity likely plays a role in each process.

## Mechanisms that affect synaptodendritic damage and network dysfunction in HAND

In vitro and in vivo models allow for probing the molecular mechanisms that underlie synaptodendritic damage and re-organization of neuronal networks in HAND. So far, a few major factors have been identified, including dysregulation of synaptic and extrasynaptic ion channels, inflammatory signaling pathways, altered synaptic actin remodeling, dysfunction of non-neuronal cells, and comorbid drug abuse. In this section, we will review recent studies on these players and pathways associated with HAND and how they are dysregulated by distinct stimuli.

### Overactivation of ionotropic glutamate receptors

NMDA receptors are ionotropic glutamate receptors that play crucial roles in neuronal function and neurotransmission [133], and they can be activated and dysregulated by HIV proteins like gp120 and tat. Gp120_IIIB_ can activate these receptors through an indirect pathway, where it first promotes secretion of the cytokine IL-1β from CNS glial cells, and IL-1β binding to interleukin-1 (IL-1) receptors on hippocampal neurons leads to phosphorylation of the NR2B subunit and activation of NR2B-containing NMDA receptors [134]. Though this early study reported cell death in their assays, which is not a key component of modern HAND, this pathway may still be relevant to synaptic function as it directly regulates NMDA receptors. Another group reported that gp120_IIIB_ directly promoted surface expression of NMDA receptors in hippocampal neurons through a protein kinase A and C-dependent phosphorylation of NR1 C-terminal residues, and these receptors were transported to gp120-stabilized lipid structures that prevented internalization and enhanced abnormal calcium bursts [135]. As all NMDA receptors have NR1 subunits, this study suggests that gp120 can activate NMDA receptors with different subunit compositions. A more recent study examined this possibility and showed that gp120_IIIB_ increased excitatory post-synaptic currents (EPSCs) from both NR2A and NR2B-containing NMDA receptors in male rat hippocampal slices, but gp120 had a more pronounced effect on NR2B-containing receptors [136]. Importantly, these recordings were taken shortly after gp120 treatments, providing further evidence that gp120 (or IL-1β)-induced NMDA receptor activation occurs prior to the homeostatic synaptic scaling adaptations discussed above. Gp120 may affect NR2B-containing NMDA receptors in particular by promoting a signaling pathway by which the pro-form of brain-derived neurotrophic factor (proBDNF) activates neuronal p75 neurotrophin receptors. The proBDNF-p75 signaling pathway contributes to long-term depression by modulating NR2B-containing NMDA receptors [137]. Gp120 may promote this signaling pathway in several ways, including by decreasing levels of the furin protease that cleaves proBDNF to its mature, neuroprotective form [138], and by increasing expression of the p75 neurotrophin receptor in the striatum [104]. In addition to its effects on long-term depression, proBDNF accumulation can also reduce hippocampal dendrite complexity and spine density through p75 neurotrophin receptor signaling [139], and these effects are mitigated in gp120-transgenic mice with a p75 knockdown [140]. Thus, gp120 can modulate NMDA receptor signaling in different types of neurons through a variety of pathways.

Adding to this complexity, evidence suggests that tat also regulates NMDA receptor functions, and tat from HIV-1 clade B may cause neurotoxicity by directly interacting with the NR1 subunit [141]. Earlier studies of tat neurotoxicity in human mixed cortical cultures reported an enhanced phosphorylation of the NR2A subunit, which led to neuronal dysfunction [142]. However, tat can also target other subunits of the NMDA receptor, as the NR2B antagonist ifenprodil prevented tat-induced synaptodendritic damage in layer I retrosplenial cortex neurons of male mice, which was associated with improved learning in a fear conditioning model [98]. In addition to these studies, a review article has documented how tat dysregulates calcium signaling through NMDA receptors as well as other calcium channels in relevant brain regions including the prefrontal cortex, hippocampus, and striatum [143]. Together, these studies suggest that NMDA receptor activation may be a point of convergence of neurotoxic signaling pathways in HAND, including for HIV proteins and inflammatory mediators.

Though brain inflammation is no longer considered a driver of cell death and apoptosis in patients on ART, chronic sub-threshold inflammation still appears to contribute to the more subtle pathology of modern HAND. For example, several studies suggest that inflammatory and synaptic systems are dysregulated in HAND models, including HIV-transgenic rats. These rats show higher markers of astrogliosis and microgliosis, dysregulation of genes associated with neurodegenerative disorders and synaptic plasticity in the hippocampus [144], and dysregulation of inflammatory genes in the prefrontal cortex, nucleus accumbens, and ventral tegmental area [145]. This chronic but subtle brain inflammation may also regulate NMDA and AMPA receptors, as previous reports show that tumor necrosis factor α (TNFα) signaling modulates these receptors functions and trafficking. Specifically, TNFα was shown to increase surface expression and activity of NMDA receptors in cultured rat hippocampal neurons [146] and AMPA receptor-mediated calcium flux [147]. Under the same treatment conditions, TNFα increased both GluR1 and GluR2-containing AMPA receptors in rat hippocampal neurons [148]. Almost in parallel, TNFα binding to TNF receptor 1 in mixed hippocampal cultures decreased GABAergic neurotransmission, which preceded changes of inhibitory synaptic proteins such as gephyrin and glutamate decarboxylase 65 [149]. Though these studies are older, they highlight important mechanisms that may underlie later synaptic remodeling and ongoing dysfunction in neuronal networks of patients with HAND.

### Synaptic scaling mechanisms

**Fig. 2:**
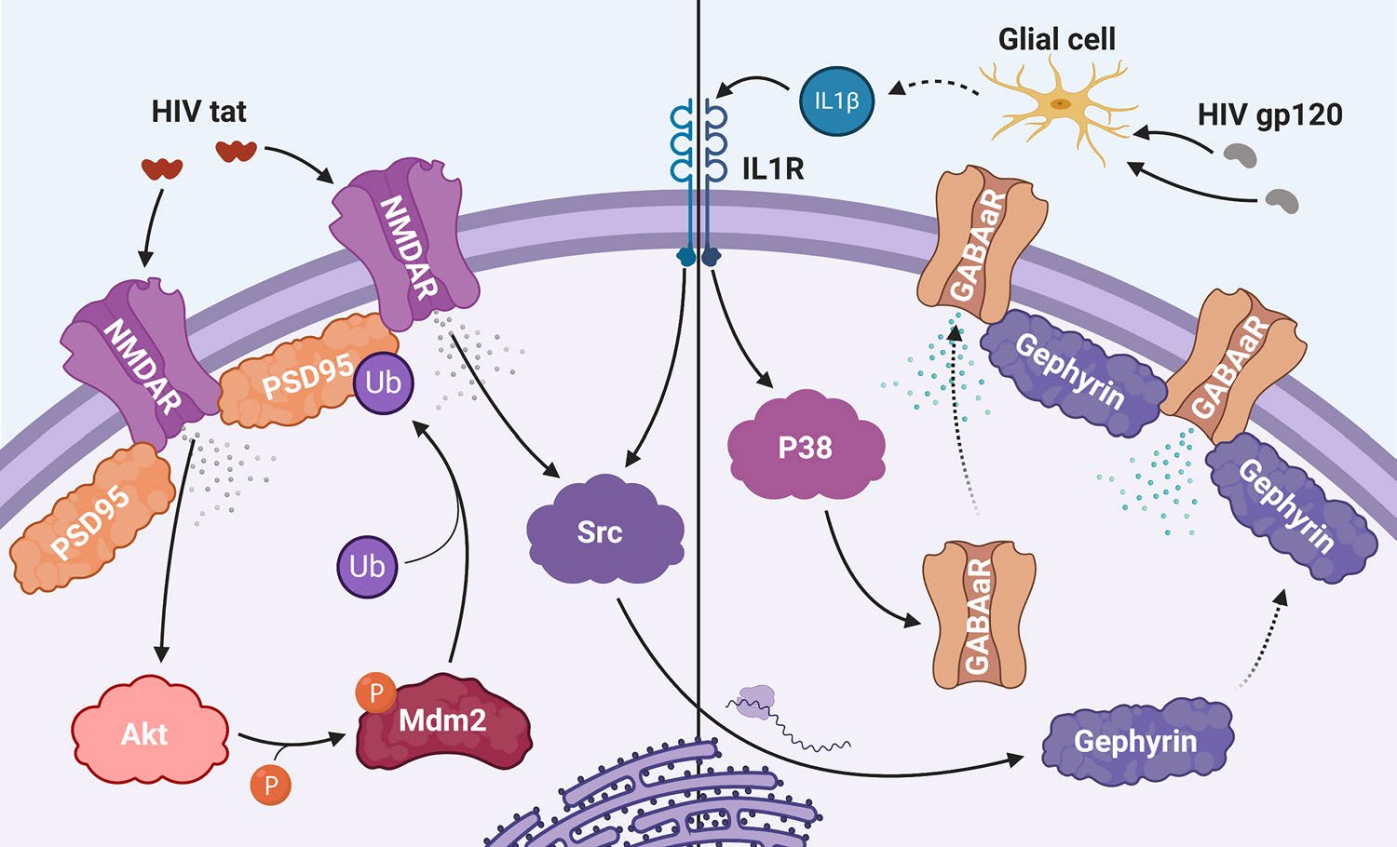
Mechanisms underlying synaptic scaling in hippocampal neurons. Synaptic scaling can serve as a feedback mechanism to prevent excitotoxicity by scaling down excitatory synapses (left panel) and increasing inhibitory synapses and transmission (right panel). Initially, HIV tat may increase NMDA receptor (NMDAR) activity by either directly interacting with the receptor or by promoting phosphorylation or membrane trafficking of intracellular NMDA receptors. Ion flux through NMDA receptors then activates the serine–threonine kinase Akt, which phosphorylates the E3 ubiquitin (Ub) ligase Mdm2. Mdm2 then ubiquitinates PSD95, an important structural component of dendritic spines, which promotes PSD95 degradation and corresponding de-potentiation of the synapse. Increased inhibition is mediated by both the initial ion influx via NMDA receptors and by gp120-mediated secretion of IL-1β from glial cells. First, IL-1β binds to neuronal IL-1 receptors (IL1R), which activates a P38-mediated pathway that leads to membrane insertion of α5-containing GABAa receptors (GABAaR) and enhanced inhibitory transmission. Additionally, IL1 receptor and NMDA receptor signaling activate the non-receptor tyrosine kinase Src, which at a later point leads to the synthesis of gephyrin, an important structural component of inhibitory synapses, its translocation to the membrane, and an increased number of inhibitory synapses

In response to the dysregulation of calcium channels and heightened vulnerability to excitotoxicity, neurons can initiate a homeostatic adaptation called synaptic scaling, which attempts to restore a balance between excitatory and inhibitory synaptic transmission. This often leads to a reduction or de-potentiation of excitatory synapses and increased inhibitory transmission in models of HAND, and several recent studies have explored the mechanisms underlying these adaptations [117] (Fig. 2). One study identified a pathway downstream of tat activation of NMDA receptors, where Akt activates the E3 ubiquitin ligase Mdm2, which then reduces the amount of NMDA receptor punctate staining in hippocampal neurons [150]. Additionally, gp120_IIIB_ upregulates Mdm2 in cortical neurons, suggesting that both HIV proteins regulate Mdm2 [151]. Mdm2 ubiquitinates and helps to degrade PSD95, a synaptic protein that provides scaffolding support for NMDA and AMPA receptors [152]. Loss of PSD95 can destabilize dendritic spines and ultimately reduce the number of excitatory synapses [153, 154]. Therefore, tat likely affects dendritic spines through this pathway, as previous evidence shows that tat promotes the degradation of PSD-95 and excitatory synapse loss but spares pro-death signaling in hippocampal neurons [155]. Gp120 and its related signaling pathways may produce similar results, as gp120 also decreases PSD-95 puncta number in cultured hippocampal neurons [134, 156], and gp120-transgenic mice showed decreased levels of NR2B and NR2A NMDA receptor subunits in synaptosome fractions from hippocampal neurons [140].

The loss of dendritic spines and excitatory potential during synaptic scaling often occurs in concert with mechanisms that enhance inhibitory neurotransmission. Two recent studies have identified mechanistic insights on how this occurs in gp120-treated hippocampal neurons. In the first, a 4-h treatment with gp120_IIIB_ increased inhibitory currents by enhancing the surface expression of neuronal α5 subunit-containing GABA_A_ receptors, and these outcomes were mediated by gp120-induced secretion of IL-1β from microglia and activation of neuronal p38 mitogen-activated protein kinase [115]. In the second study, a 24-h treatment with gp120_IIIB_ increased the number of gephyrin-labeled inhibitory synapses, which also involved IL-1β activation of p38 in addition to NMDA receptor and Src kinase-mediated protein synthesis [114]. Tat exposure can also affect hippocampal inhibitory neurotransmission, as another study shows that 24-h tat treatment increases gephyrin-labeled inhibitory synapses and down-regulates PSD-95, which first involved neuronal uptake of tat and subsequent activation of NR2A-containing NMDA receptors and Ca^2+^/calmodulin-dependent protein kinase II, and later required NR2B-containing NMDA receptors [157]. Importantly, these adaptations do not occur the same way in different neuronal networks, as tat attenuated GABA exocytosis from neocortical synaptosomes [158], dysregulated GABAergic transmission in human differentiated neuronal cultures by reducing potassium/chloride cotransporter 2 (KCC2) levels [110], and decreased miniature inhibitory post-synaptic currents in mouse prefrontal cortex neurons by impairing CB1 receptor signaling [159]. Notably, tat treatment in combination with morphine further reduced both the spontaneous and miniature inhibitory post-synaptic currents in mouse striatal slices, and this combined effect was blocked by the opioid antagonist naloxone [111]. The studies in this section provide valuable insights on why different brain areas are more vulnerable to HIV neurotoxins and lend additional support to the hypothesis that HAND is driven by synaptodendritic damage and network dysfunction in specific brain regions.

### Rearrangement of actin filaments

Dendritic spines are rich in actin. A balance between synaptic actin polymerization and depolymerization regulates their morphology and maintenance [160]**.** These processes are often disrupted in neurologic disorders, including HAND [37, 161]. Recent studies in animal models have shown that spine alterations in the prefrontal cortex of male HIV-transgenic rats involve an actin depolymerization pathway regulated by the small GTPase Ras-related C3 botulinum toxin substrate 1 (Rac1) [84]. Normally, Rac1 activation leads to phosphorylation and activation of p21-activated kinase 1 (PAK1), which phosphorylates LIM kinase 1 (LIMK1), an inhibitor of the actin severing protein cofilin [162, 163]. However, activation of Rac1 and PAK1 is reduced in frontal cortex of HIV-transgenic rats [84], suggesting an increase of cofilin-mediated actin severing that could drive loss of dendritic spines in this region. Intriguingly, this Rac1 pathway is activated by the CXCL12/CXCR4 chemokine axis, and CXCL12 injection into the lateral ventricle of HIV-transgenic rats reversed dendritic spine deficits in layer II/III prefrontal cortex neurons and improved performance in an operant set-shifting task [84]. Both effects depend on Rac1 signaling, suggesting that targeting synaptic actin modulators may restore neuronal network functions and improve cognitive performance in HAND. A re-organization of actin filaments may also participate to synaptic scaling adaptations of hippocampal neurons exposed to tat. One study showed that tat-mediated activation of NMDA receptors stimulates Ras homolog family member A (RhoA) and Rho-associated protein kinase (ROCK), which scale down calcium influx from NMDA receptors by altering actin dynamics [164]. Interestingly, RhoA and ROCK signaling were also previously reported to inactivate cofilin through phosphorylation by LIMK1 [165], suggesting that inhibiting actin turnover in hippocampal neurons suppresses calcium influx from NMDA receptors. However, this is likely an oversimplification as studies in CXCL12-treated rats show that Rac1-mediated spine stabilization in the prefrontal cortex is a critical contributor to improved cognitive performance [84], indicating that these stabilized spines effectively integrate into the local neuronal network. Incidentally, CXCL12 has additional neuroprotective effects both in vivo and in vitro [166–168]. It is difficult to make direct comparisons between the tat and CXCL12 studies, since they examine different types of neurons in different conditions. Future studies should illuminate how pathways that promote cofilin phosphorylation produce different outcomes in relevant brain regions. Furthermore, it should also be noted that past studies have reported neurotoxic effects of this chemokine under specific culture conditions [169]. These effects are likely due to glial involvement and cleavage of CXCL12, which result in a product unable to stimulate CXCR4 and signals via CXCR3 instead [62, 170].

### Dysfunction of astrocytes

Astrocytes regulate synaptic glutamate levels by taking up excess glutamate through synaptically localized glutamate transporters, and this process can be dysregulated by both inflammatory mediators and HIV proteins [171, 172]. Indeed, reduced expression of excitatory amino acid transporter 2 (EAAT2) has been reported in the striatum of male gp120-transgenic mice—an event associated with decreased glial and synaptic uptake of glutamate in the striatum but not in the hippocampus, suggesting that striatal astrocytes are vulnerable to gp120 [173]. Studies in other HAND models have identified additional mechanisms that could interfere with glutamate transport both in vitro and in vivo. These include the cytokine oncostatin M, which is secreted by microglia in primary cortical cultures exposed to ecoHIV [174] and the astrocyte elevated gene-1 (AEG-1), which is induced by IL-1β and TNFα and upregulated in the brains of HAND patients and tat-transgenic mice [175]. Both oncostatin M and AEG-1 lower EAATs expression via a JAK/STAT and NF-κB pathway, respectively [174, 175]. Interestingly, AEG-1 is implicated in several neurologic disorders and is increased in aged individuals [176].

Some ART medications may also disrupt astrocyte clearance of glutamate at therapeutic concentrations, as studies show that the protease inhibitors amprenavir and lopinavir reduce EAAT2 levels and function in primary human astrocytes [177]. Thus, the loss of neuronal support described above may be compounded by ART [178] as well as changes in glial cell metabolism [179]. Astrocytes exposed to HIV tat and cocaine shift their metabolic status from glucose oxidation to fatty acid oxidation, which is associated with a reduced production of lactate [180, 181]. Neurons use lactate as a major metabolic substrate, so this metabolic shift limits neuronal bioenergetic potential. Subtle inflammation may also regulate astrocyte bioenergetics, as IL-1β increases astrocyte metabolic activity and expression of genes related to mitochondria and inflammation, and these changes are associated with secretion of additional proinflammatory molecules [182]. Furthermore, HIV as well as select antiretroviral drugs promote endoplasmic reticulum stress and mitochondrial dysfunction [183], which highlights additional pathways that can contribute to metabolic dysfunction. Thus, the loss of support from astrocytes on multiple fronts is an important component of modern HAND, specifically considering that some antiretrovirals may promote these outcomes.

### Opioids and other drugs of abuse

**Fig. 3:**
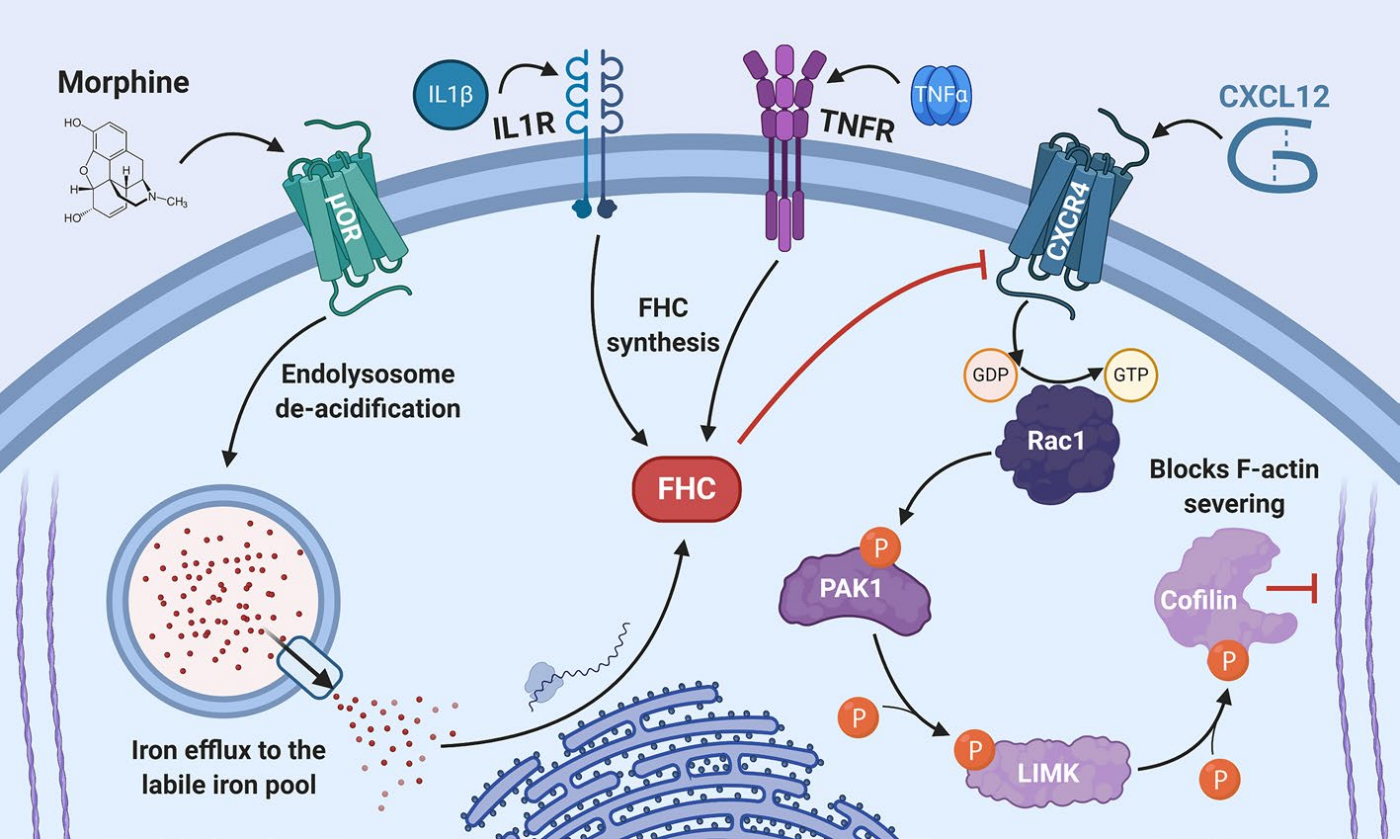
Opioid and inflammatory regulation of ferritin heavy chain and corresponding effects on CXCR4 signaling and dendritic spines. Our studies indicate that µ-opioid agonists and inflammatory signaling pathways in cortical neurons converge to upregulate ferritin heavy chain (FHC), an iron storage protein that also inhibits CXCR4 signaling and produces corresponding dendritic spine deficits. Morphine activation of µ-opioid receptors (µOR) promotes de-acidification of endolysosomes and release of endolysosomal iron. This increases free labile iron in the cytosol, to which neurons respond by post-transcriptionally upregulating FHC protein levels. In the same system, FHC is also upregulated by inflammatory signaling pathways, including downstream of IL-1β/IL1 receptor (IL1R) and TNFα/TNF receptor (TNFR) signaling. In each case, FHC inhibits activation of CXCR4 via its natural ligand CXCL12, which prevents CXCR4-induced stabilization of dendritic spines. Normally, CXCL12/CXCR4 signaling enhances dendritic spine density by increasing the amount of activated, GTP-bound Rac1, which promotes a cascading phosphorylation of PAK1, LIMK, and the actin severing protein cofilin. Phosphorylation of cofilin blocks its ability to break down synaptic actin, which stabilizes dendritic spines and also reverses spine deficits in HIV-transgenic rats

Comorbid drug abuse is common in patients with HAND, and recent studies have uncovered some of the mechanisms by which drugs of abuse contribute to synaptodendritic damage in HAND models. For example, our studies in cortical neurons showed that morphine activation of µ-opioid receptors inhibits signaling of the dendritic spine promoting chemokine receptor CXCR4 through a mechanism that required upregulation of ferritin heavy chain protein [184] (Fig. 3). Although ferritin heavy chain is well known as a subunit of the iron storage protein ferritin, it also interacts with and inhibits CXCR4 and possibly other chemokine receptors [185]. Follow-up studies produced similar findings in clinical samples from human HAND patients and opioid/poly drug users and demonstrated that morphine-mediated dendritic spine deficits in cultured rat cortical neurons required upregulation of ferritin heavy chain in these cells [122]. Ferritin heavy chain was also upregulated by the inflammatory mediators TNFα and IL-1β [90]. Interestingly, morphine-mediated ferritin heavy chain upregulation and subsequent dendritic spine deficits occurred via a pathway that modulates endolysosomal iron stores [186]. These studies indicate that morphine and potentially other µ-opioid agonists produce synaptodendritic damage by dysregulating neuronal iron metabolism. Interestingly, another group reported that NMDA receptor activation in mouse hippocampal neurons also promoted lysosomal iron efflux into the cytoplasm, which stimulated a PKC and Src kinase-mediated pathway that downregulated the NR2A NMDA receptor subunit and reduced neuronal excitability [187]. Additional studies have shown that morphine in combination with HIV neurotoxins produces synaptodendritic damage in the mouse striatum, which also involves µ-opioid receptor signaling, activation of NMDA and AMPA receptors, and release of calcium from intracellular stores [121, 188]. A review that catalogues opioids effects on dendritic spines and synaptodendritic damage in greater detail has recently been published [37].

Comorbid use of stimulant drugs may also contribute to synaptodendritic injury and altered excitability of neurons in specific brain regions. One group showed that cocaine treatment enhanced excitability and calcium spikes of layer V and VI rat medial prefrontal cortex neurons exposed to tat, and tat’s effects were blocked by the L-type calcium channel antagonist diltiazem [189]. A follow-up study in male HIV-transgenic rats reported increased expression of the L-type channel Cav1.2-α1c in the same layers of the medial prefrontal cortex, and forced abstinence after cocaine self-administration resulted in increased excitability of these neurons that was again mitigated by diltiazem [190]. There is also evidence that methamphetamine contributes to synaptodendritic injury in animal models of HAND, as studies examining male and female tat-transgenic mice [113] and male HIV-transgenic rats [112] report region-specific changes of synaptic and neuroplasticity markers in methamphetamine-treated animals. Furthermore, gene expression changes were reported in the mPFC of HIV-transgenic rats that self-administered METH indicative of neuroinflammation and neuronal injury [191]. Gene expression studies also show that gp120-transgenic mice with and without methamphetamine treatment had significant alterations of genes involved in glutamatergic and GABAergic neurotransmission (including subunits of GABA, AMPA, and NMDA receptors) in treated animals [192]. These findings are in line with data from human subjects [39, 40, 58]. Although each of the drugs discussed in this section can activate different pathways and produce different outcomes, evidence suggests that they all promote synaptodendritic injury or dysregulate neuronal excitability, which can help to explain why they are associated with cognitive impairment in HAND.

## Conclusions and future challenges

The drivers of HAND pathology in treated patients appear to be both complex and subtle. A few underlying themes promoted by both host and viral factors, such as cytokines, inflammatory mediators, and HIV toxins, fuel the pathology leading to CNS, vascular, and metabolic dysfunction. These insults are often exacerbated by comorbidities like substance abuse and aging as well as select ART regimens. Neurons in some brain regions can adapt to the continued presence of sub-threshold insults, but others seem more vulnerable to synaptodendritic damage and network alterations. Thus, this warrants continued exploration of the mechanisms that produce the subtle synaptodendritic damage underlying cognitive impairment in HAND.

As published neuropathology studies that examined synaptic damage used brain tissue from patients with uncontrolled infections or with AIDS, we lack a complete picture of how synaptodendritic damage presents in today’s treated patients. Obtaining enough tissue for these studies to be meaningful will be challenging, as treated patients live much longer than the patients examined in older studies. In the absence of updated neuropathology studies, much of the knowledge on modern-day HAND will have to rely on advanced neuroimaging techniques. Though these techniques cannot directly measure synaptic damage in the brain and have produced conflicting results depending on the specific imaging approach, they can provide insights on macro-level brain alterations that can complement other powerful approaches like gene array studies (currently limited by brain tissue availability), and/or investigations in animal models. Moreover, investigations on neuronal network alterations in HAND can identify changes in neuronal functions in the absence of structural deficits. Thus, functional connectivity studies in HAND patients and animal models can inform examinations of how HIV infection alters brain function even when the infection is controlled. This is important, because HIV-induced changes in synaptic function may precede structural damage or adaptations in the same network. Targeting these upstream pathways could provide new approaches to reverse synaptodendritic damage and cognitive impairment in treated patients. Lending further support to this approach, several studies demonstrate that dendritic spine deficits and cognitive impairment are reversible in animal models of HAND. However, one of the major challenges in this line of study is to determine how factors that contribute to HAND work together to disrupt neuronal circuits. Another challenge will be teasing apart HIV-related alterations of network activity and the resulting neuronal adaptations at a structural and functional level. As certain brain regions appear more adaptive than others, a third challenge will be to determine what makes a brain region or neuronal network vulnerable to HIV-specific insults. Future studies that address these points will provide critical information about why HAND persists in treated patients.

Finally, the known approaches to reverse dendritic spine deficits in animal models are a proof of concept, but additional research is required to translate this information into safe and effective treatments for humans. This is especially true considering previous clinical trial failures of compounds designed to target NMDA receptors [193]. Recent studies have demonstrated several paths forward, but thorough mechanistic examinations will be required to identify targets that can be therapeutically modulated without detrimental off-target effects. These studies will benefit from experimental approaches that integrate examination of molecular mechanisms and transcriptomic profiles with changes in neuronal structure and function and behavioral modifications in animal models. An integrated experimental framework will increase scientific rigor and may be more likely to produce insights that translate to the clinic. As new approaches and experimental tools enhance our ability to more precisely examine specific brain circuits [194], future research has the potential to study HAND in a way that respects its current presentation as a complex disorder.
